# Association between maternal breastfeeding and risk of systemic neoplasms of offspring

**DOI:** 10.1186/s13052-022-01292-9

**Published:** 2022-06-16

**Authors:** Qin-Qin Gong, Dan-Dan Quan, Chong Guo, Chao Zhang, Zhi-Jun Zhang

**Affiliations:** 1 Center of Women’s Health Sciences, Taihe Hospital, Hubei University of Medicine, Shiyan, 442000 China; 2 Department of Obstetrics and Gynecology, The People’s Hospital of China Three Gorges University, The First Hospital of Yichang, Yichang, 443000 China; 3 Department of Gynaecology and Obstetrics, Taihe Hospital, Hubei University of Medicine, Shiyan, 442000 China; 4 Center for Evidence-Based Medicine and Clinical Research, Taihe Hospital, Hubei University of Medicine, No.32, South Renmin Road, Shiyan, 442000 China; 5 Center for Reproductive Medicine, Taihe Hospital, Hubei University of Medicine, No.32, South Renmin Road, Shiyan, 442000 China

**Keywords:** Breastfeeding, Childhood cancer, Acute lymphocytic leukemia, Acute myeloid leukemia, Non-lymphocytic leukemia

## Abstract

**Background:**

Breastfeeding might prevent childhood cancer by stimulating the immune system.

**Methods:**

The following databases, including PubMed, Embase, and Cochrane Library, were searched from inception to January 10, 2021.

**Results:**

In dose-dependent manner, there was a statistically significant inverse association between any breastfeeding and the incidence of childhood cancer. There was no evidence that breastfeeding was inversely related to childhood cancer of the skeletal, reproductive, or sensory systems. However, breastfeeding was inversely associated with the incidence of hematological malignancies and cancers of the nervous and urinary systems. Among hematological malignancies, the relationship was significant for acute lymphocytic leukemia (ALL) and acute myeloid leukemia (AML), but not for acute non-lymphocytic leukemia (ANLL), Hodgkin’s lymphoma (HL), or non-HL.

**Conclusions:**

The evidences demonstrated that breastfeeding have a potential protective role in preventing selective childhood cancer growth, especially for ALL, AML, cancer of nervous and urinary systems. This study recommended that breastfeeding be extended for as long as possible or maintained for at least 6 months to prevent selective childhood cancer growth.

**Supplementary Information:**

The online version contains supplementary material available at 10.1186/s13052-022-01292-9.

## Background

The incidence and mortality associated with childhood cancer is increasing sharply in developed and developing countries [1]. The most common childhood cancers include acute leukemia at 26.3%, especial for acute lymphoblastic leukemia (ALL) [2], central nervous system tumors at 17.6%, and lymphoma at 14.6% of all cancers [3].

Breastfeeding is the major food for newborn babies, who receive almost all essential needs to meet the requirements of growth and development [4]. It has become a universal phenomenon for babies to be fed with formula in recent years; nevertheless, components of formula are different from those of breastfeeding. Breastfeeding has components not found in formula, including active hormones and peptides, all of which play essential roles in development during the newborn period and infancy. A researcher suggested that breastfeeding might help prevent childhood cancer by stimulating the immune system. To date, several lines of evidence suggest that breastfed babies are healthier, with benefits of increased immunity and intelligence, lower incidence of sudden infant death, childhood obesity, and allergies [5–7]. There was a hypothesis that breastfeeding might help protect against several childhood diseases, not limited to ALL [8].

There were eight studies reporting the results of meta-analyses on the relationship between breastfeeding and childhood cancer [4, 9–15]. The relationship of breastfeeding and hematological malignancy-related diseases was confirmed by several studies of these studies [4, 9–12, 14, 15]. Two studies expanded the scope of research to the relationship between breastfeeding and central nervous system diseases [13, 14]. There was little evidence to suggest that breastfeeding was significantly associated with acute non-lymphoblastic leukemia (ANLL) [16–18], non-Hodgkin’s lymphoma (NHL) [9, 16, 17, 19, 20], central nervous system cancers, malignant germ cell tumors, juvenile bone tumors and other solid cancers. None of these studies mentioned breastfeeding and other systemic diseases and the influence of breastfeeding on childhood cancer. Moreover, a clear association between modes of breastfeeding (or formula) and risk of childhood cancer had not been explored in detail in previous studies. The World Health Organization (WHO) suggested that breastfeeding had additional health benefits that extend into adulthood, and it recommended maintaining breastfeeding for 2 years or longer [21]. With respect to the duration of breastfeeding, there were controversy among researchers and no consensus had yet been reached. Given the lack of comprehensive and systematic research confirmed by reviewing the literature, this meta-analysis was conducted to comprehensively explore the association of breastfeeding and childhood cancer, involving several countries, modes of breastfeeding, various feeding durations, and systemic diseases of childhood.

## Methods

### Search strategy

PubMed and Embase were systematically searched for relevant studies that met our eligibility criteria. The literature search was carried out on January 10, 2021 to identify published studies on the relationship of breastfeeding and cancer in children. Two authors were responsible for screening the studies to obtain full manuscripts, as well as the titles and abstracts. To ensure completeness and accuracy of this meta-analysis and systematic review, two reviewers participated in the entire literature search process without interfering with one another. The search terms for this study were as follows: “Childhood”, “Children”, “Child”, “Neoplasms”, “Cancer”, “Tumour”, “Neoplastic”, “Malignancy”, “Bottle-feeding”, “Breastfeeding”, “Infant nutrition”, “Perinatal”, and “Milk”, with language and publication status restrictions. The Preferred Reporting Items for Systematic Reviews and Meta-Analyses (PRISMA) was followed [22].

### Eligibility criteria

Studies were included in the meta-analysis if they met the following criteria: (1) the study was published in English; (2) the exposure of interest was breastfeeding; (3) the outcome was childhood cancer; (4) the age of included population was less than 18 years old; and (5) the estimates of the relative odds ratio (OR) with 95% confidence interval (CI) were reported.

Exclusion criteria were as follows: (1) duplicated reports; (2) defective study designs; (3) incomplete data and uncertain outcome effects; and (4) incorrect statistical methods or those that could not be amended, could not be provided, or could not be converted into OR.

### Data extraction

The following data were extracted from each study: country of the patients, individual ages and average ages, types of cancer (e.g., hematological malignancies including lymphoma and leukemia, and cancers of the nervous, motor, urinary, reproductive, and sensory systems), the modes of breastfeeding (e.g., exclusive breastfeeding, mixed breastfeeding, and bottle feeding or formula feeding; non-exclusive breastfeeding including mixed and formula feeding; the same classification applied for non-mixed and -formula feeding), feeding duration, and the number of patients in the breastfeeding and control. Adjustment factors and other directly extractable data were first extracted for each study. The durations of breastfeeding were classified as either past breastfeeding or never breastfed, greater than or equal to 6 months or less than 6 months, and greater than or equal to 12 months or less than 12 months.

### Validity assessment

The Newcastle-Ottawa Scale (NOS) was used to assess quality of included studies in this meta-analysis and systematic review [23]. NOS includes case definition, representativeness of the cases, selection of controls, definition of controls, comparability of cases and controls, ascertainment of exposure, same method of ascertainment for cases and controls, and no response rate. Each asterisk in the table represents a point, and the final score is the sum of the points.

### Statistical analysis

The effect of breastfeeding on childhood cancer was analyzed using OR and 95%CI as the effect measure. Between-study heterogeneity [22] was assessed using Cochran’s Q and I^2^ statistics. Initial analyses with I^2^ < 40% were performed using a fixed-effects model; otherwise, a random effects model was adopted. Potential confounders, including the modes of breastfeeding (exclusive, mixed, and formula with different durations of breastfeeding), duration of breastfeeding (including ≥1 month vs. < 1 month, ≥6 months vs. < 6 months, and ≥ 12 months vs. < 12 months), different countries, and cancers of different systems were regarded as the main sources of heterogeneity. Subgroup and sensitivity analyses were employed. We used the one-stage robust error meta-regression model to establish the potential dose-response relationship between duration of breastfeeding and risk of cancer [24, 25]. This was a one-stage method that treated each study as a cluster combined with robust error estimation as a solution to deal with potential correlations within each study [26]. The restricted cubic spline function with three auto-generated knots was used to fit the potential non-linearity trends [27]. The remr command of Stata software was used to run the dose-response meta-analyses [28]. All statistical analyses were performed using RevMan 5.3 and STATA 15.0.

## Results

### All studies

**Fig. 1:**
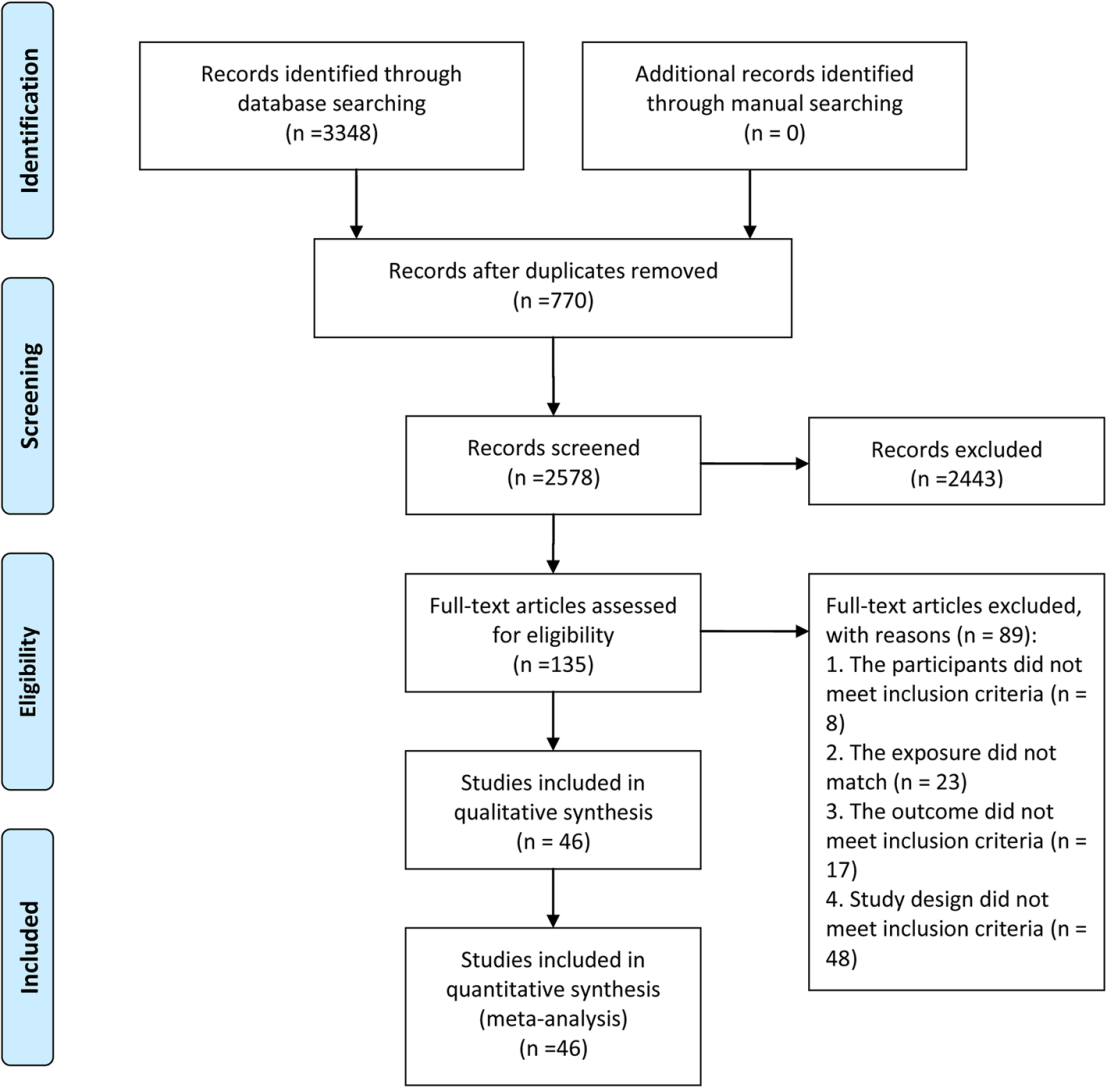
Flow diagram of study selection

A total of 3348 studies were obtained through a search of electronic databases, and 46 studies, including 20,066 cases and 448,661 control individuals, met our eligibility criteria. A total of 770 studies were removed for being duplicates, 2443 for being irrelevant (as determined by reading the title and abstracts), and 89 for reasons determined by reading the full text. Studies also were excluded if data could not be extracted or could not be obtained by contact with the author (see Additional file 1: Table 1). A flow diagram was shown in Fig. 1.

Of these 46 studies [8, 9, 16–20, 29–67], 24 studies published between 1988 to 2017 [8, 16, 18–20, 29, 31–33, 36, 39, 40, 42, 43, 47, 52–56, 60, 62, 64, 66] discussed hematological malignancies, including 19 studies [8, 16, 18, 19, 29, 32, 36, 39, 40, 42, 43, 52–56, 60, 62, 66] of leukemia (ALL, acute myeloid leukemia (AML) and ANLL). 5 studies [9, 16, 18–20] reported relevant data about lymphoma (Hodgkin’s lymphoma (HL) and non-Hodgkin’s lymphoma (NHL)). All included studies in this meta-analysis questioned the influence of the modes and duration of breastfeeding on childhood cancer. The main features of the studies were displayed in Additional file 1: Table 1. The reasons for excluding 96 studies in detail were displayed in Additional file 1: Table 2. All funnel plots were symmetrical, suggesting absence of bias.

### Whether breastfeeding impacts the incidence of childhood Cancer

**Fig. 2:**
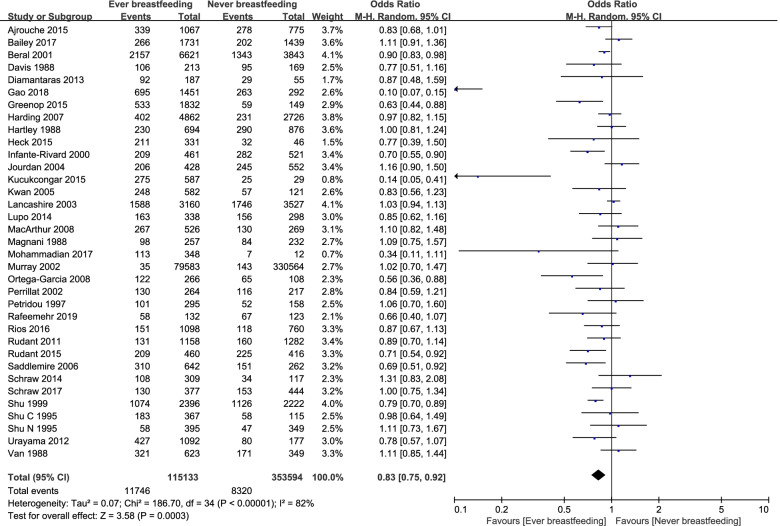
Forest plot demonstrating the incidence of children with cancer in terms of ever breastfeeding versus never breastfeeding

Thirty-five studies [8, 9, 16–18, 20, 29, 31–43, 46, 47, 49, 50, 52–54, 56, 59, 60, 62, 64–67] explored whether breastfeeding impacted the incidence of childhood cancer. Compared to children that were never breastfed, the odds ratio of ever having been breastfed was 0.83 (95%CI, 0.75–0.92) with respect to childhood cancer (Fig. 2), suggesting that ever having been breastfed was associated with a significant reduction in the incidence of childhood cancer.

### Modes of breastfeeding

**Fig. 3:**
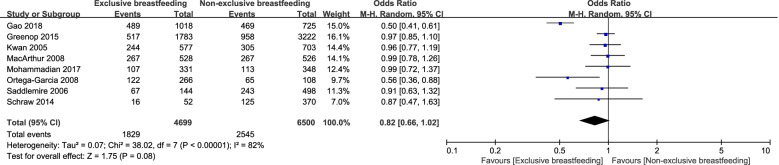
Forest plot demonstrating the incidence of children with cancer in terms of exclusive versus non-exclusive breastfeeding

With respect to the various modes of breastfeeding, there was no significant difference in incidence of childhood cancer between exclusive and non-exclusive breastfeeding (OR = 0.82, 95%CI, 0.66–1.02) (Fig. 3 and Table 1). However, the others modes of breastfeeding and the incidence of childhood cancer (mixed vs. non-mixed breastfeeding (OR = 0.95, 95%CI, 0.91–1.00) and formula vs. non-formula feeding (OR = 1.38, 95%CI, 1.00–1.92)) showed significant differences. Mixed breastfeeding vs. formula (OR = 0.75, 95%CI, 0.53–1.07), exclusive breastfeeding vs. formula (OR = 0.54, 95%CI, 0.23–1.25), and the comparison of exclusive vs. mixed (OR = 1.01, 95%CI, 0.92–1.10) showed no significant differences in the incidence of childhood cancers. All details regarding modes of breastfeeding associated with the incidence of childhood cancer were shown in Table 1.

**Table 1:** Subgroup analyses of different outcome indicators

Outcome indicators	Number of studies	Sample size	OR, 95%CI	P for OR	I^**2**^	I^**2**^ for P
**Childhood cancer (Ever vs. Never)**	35	468,727	0.83 [0.75, 0.92]	0.0003	82%	*P* < 0.00001
**Duration**						
Duration ≥1 month vs. < 1 month	11	25,609	0.75 [0.63, 0.89]	0.001	80%	*P* < 0.00001
Duration ≥6 months vs. < 6 months	32	41,813	0.82 [0.74, 0.90]	0.0001	62%	*P* < 0.00001
Duration ≥12 months vs. < 12 months	13	8490	0.82 [0.65, 1.04]	0.09	68%	*P* < 0.0002
Exclusive ≥6 months vs. 6 months	4	3243	0.98 [0.75, 1.28]	0.88	45%	0.14
**Country developed vs. developing**	31	465,845	0.89 [0.83, 0.95]	0.001	55%	0.0002
**Gender female vs. male**	29	467,962	0.96 [0.92, 1.01]	0.13	0%	0.71
**High quality studies**	24	33,407	0.78 [0.67, 0.91]	0.002	88%	*P* < 0.00001
**The mode of breastfeeding**						
Exclusive vs. Mixed and Formula	8	11,199	0.82 [0.66, 1.02]	0.08	82%	*P* < 0.00001
Mixed vs. Formula and Exclusive	10	28,166	0.95 [0.91, 1.00]	0.07	41%	0.8
Formula vs. Mixed and Exclusive	8	26,165	1.38 [1.00, 1.92]	0.05	95%	*P* < 0.00001
**Exclusive vs. Mixed**	7	8865	0.99 [0.90, 1.08]	0.82	0%	0.99
Exclusive vs. Mixed 0–6 months	3	3234	0.96 [0.80, 1.15]	0.68	45%	0.14
Exclusive vs. Mixed 6–12 months	1	251	1.08 [0.65, 1.81]	0.76	NA	NA
Exclusive vs. Mixed > 12 months	1	142	0.78 [0.37, 1.64]	0.52	NA	NA
**Exclusive vs. Formula**	5	5764	0.54 [0.23, 1.25]	0.15	96%	*P* < 0.00001
**Mixed vs. Formula**	7	22,361	0.75 [0.53, 1.07]	0.11	95%	*P* < 0.00001
Mixed vs. Formula < 1 month	1	7891	1.02 [0.91, 1.01]	0.75	NA	NA
Mixed vs. Formula ≥1 month	1	7650	1.01 [0.91, 1.14]	0.84	NA	NA
**Type of cancer**						
**Hematologic malignancies**	24	445,481	0.82 [0.71,0.94]	0.005	85%	*P* < 0.00001
**Leukemia**	19	437,644	0.89 [0.82, 0.97]	0.009	49%	0.008
ALL	19	435,166	0.90 [0.85, 0.95]	0.0003	55%	0.002
AML	3	8761	0.79 [0.66, 0.94]	0.009	62%	0.07
ANLL	3	694	1.10 [0.68, 1.80]	0.69	0%	0.95
**Lymphoma**	5	10,443	0.93 [0.79, 1.09]	0.36	0%	0.72
HL	4	8270	0.89 [0.68, 1.15]	0.36	0%	0.63
NHL	5	9318	0.96 [0.79, 1.17]	0.67	0%	0.78
**Nervous system**	9	19,923	0.72 [0.54, 0.96]	0.02	89%	*P* < 0.00001
**Skeletal system**	1	636	0.85 [0.62, 1.16]	0.30	NA	NA
**Urinary system**	1	904	0.69 [0.51, 0.92]	0.01	NA	NA
**Reproductive system**	1	744	1.11 [0.73, 1.67]	0.63	NA	NA
**Sensory system**	1	337	0.77 [0.39, 1.50]	0.44	NA	NA
**Three largest studies**	3	21,769	0.90 [0.79, 1.04]	0.16	84%	0.002

*ALL* Acute lymphocytic leukemia, *AML* Acute myeloid leukemia, *ANLL* Acute non-lymphocytic leukemia, *HL* Hodgkin’s lymphoma, *NHL* Non-Hodgkin’s lymphoma

### Duration of breastfeeding

**Fig. 4:**
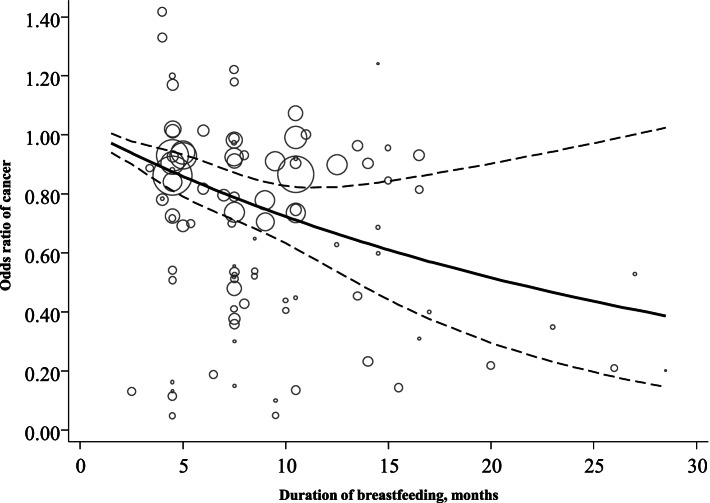
Dose-response relationships between duration of breastfeeding and risk of cancer

We observed a non-linear dose-response relationship between duration of breastfeeding and risk of cancer (P for non-linear test < 0.01). The risks of cancer were inversely associated with the duration of breastfeeding: as duration of breastfeeding increased, the odds of cancer significantly decreased (Fig. 4).

Grouped based on the duration of breastfeeding, there were cut-off points at months 1, 6, and 12 for exclusive breastfeeding. Table 1 indicates that the length of breastfeeding was significantly related to the incidence of cancer, especially for long-term breastfeeding (≥1 month vs. < 1 month (OR = 0.75, 95%CI, 0.63–0.89), and ≥ 6 months vs. < 6 months (OR = 0.82, 95%CI, 0.74–0.90). However, no difference was observed for the comparison between 12 months of breastfeeding (≥12 months vs. < 12 months (OR = 0.82, 95%CI: 0.65–1.04).

Regarding exclusive breastfeeding for 6 months, four studies [33, 39, 47, 52] were included in this meta-analysis; there was no significant difference between groups in terms of incidence of childhood cancer (≥6 months vs. < 6 months, OR = 0.98 95%CI, 0.75–1.28). Compared with mixed breastfeeding, there were no data for incidence of childhood cancer for 6–12 months and ≥ 12 months of exclusive breastfeeding, with the exception of a comparison of exclusive breastfeeding for 0–6 months vs. mixed breastfeeding (OR = 0.92, 95%CI, 0.78–1.08). Significant meaningful data were not found (Table 1**)**.

### Studies in developed countries

Because of various economic levels among countries, breastfeeding conditions were inconsistent, as was the incidence of childhood cancer. The data of 31 studies were analyzed [8, 9, 16, 17, 19, 20, 32, 34–36, 38–43, 46, 47, 49, 50, 52–54, 56, 60, 62, 64–68] from developed countries, and there was a significant inverse relationship between any breastfeeding and childhood cancer (OR = 0.89, 95%CI, 0.83–0.95) (Table 1).

### Gender

Only the differences with respect to gender in the incidence of childhood cancer had been compared, and there were no data on the prevalence of males and females in the various breastfeeding conditions. Twenty-nine studies [9, 17, 20, 29–35, 37, 39–43, 45–48, 51–53, 56–58, 63–65], involving 467,962 individuals were included in this meta-analysis and systematic review; the result of females vs. males (OR = 0.96, 95%CI, 0.92–1.01) suggested no significant difference between the sexes with respect to incidence of childhood cancer (Table 1).

### The largest studies and high-quality studies

The studies with more than 1000 cases were considered the largest studies [4]. The largest study [69], conducted in the United Kingdom by the UK Childhood Cancer Study Investigators and published in 2001, included 2157 cases. For those who were ever breastfed compared to those who were never breastfed (OR = 0.89, 95%CI, 0.84–1.00) there was a weak connection of borderline statistical significance to the effect that any breastfeeding was associated with a slight reduction in the incidence of childhood cancer. This study also included a subgroup analysis of the duration of breastfeeding. Only 1–6 months of breastfeeding (OR = 0.88, 95%CI, 0.79–0.98) showed a statistically significant inverse relationship with the incidence of childhood cancer. According to a meta-analysis by Lancashire et al. [54] in the United Kingdom, published in 2003 and including 1588 cases, neither ever being breastfed (compare with never) (OR = 1.01, 95%CI, 0.91–1.11), nor the duration of breastfeeding (< 1 month: OR = 1.05, 95%CI, 0.91–1.22, 1–6 months: OR = 0.97, 95%CI, 0.87–1.09, > 6 months: OR = 1.04, 95%CI, 0.85–1.27) showed any correlation with the risk of childhood cancer. A study of 1074 participants conducted in the United States and published in 1999 by Shu et al. [62] also investigated the influence of breastfeeding on the incidence of childhood cancer, and evaluated whether long breastfeeding duration was more protective. Their results were statistically significant and indicated that any breastfeeding (whatever form and regardless of length of breastfeeding) decreased the incidence of childhood cancer (OR = 0.79, 95%CI, 0.70–0.91); the effects of more than 6 months of breastfeeding (OR = 0.70, 95%CI, 0.59–0.82) were more pronounced for the incidence of childhood cancer in the three largest studies.

These three studies were analyzed separately by our reviewers without interfering with one another [54, 62, 69]. We found no remarkably significant inverse association between any breastfeeding and childhood cancer (OR = 0.90, 95%CI, 0.79–1.04) (Table 1**)**. However, the common comparison was 6 months or more of breastfeeding compared with a shorter duration in the three largest studies; the results demonstrated weak evidence of a protective association between breastfeeding for duration of 6 months or more and the incidence of childhood cancer (OR = 0.93, 95%CI, 0.86–1.01).

NOS for assessing the quality of included studies was shown in Additional file 1: Table 3. The scores ranged from 4 to 9. In total, we obtained 32 high-quality studies [8, 9, 18, 20, 29–38, 40, 44–48, 50, 52–54, 57–63, 66] with scores greater than or equal to 6. It included 24 studies [8, 9, 18, 20, 29, 31–33, 35–38, 40, 46, 47, 50, 52–54, 59, 60, 62, 65, 66] in an evaluation of whether breastfeeding impacted childhood cancer, and it found a significant relationship between duration of ≥1 month vs. < 1 month and the incidence of childhood cancer (OR = 0.75, 95%CI, 0.63–0.89) (Table 1).

### Different childhood cancers

#### Hematological malignancies

**Fig. 5:**
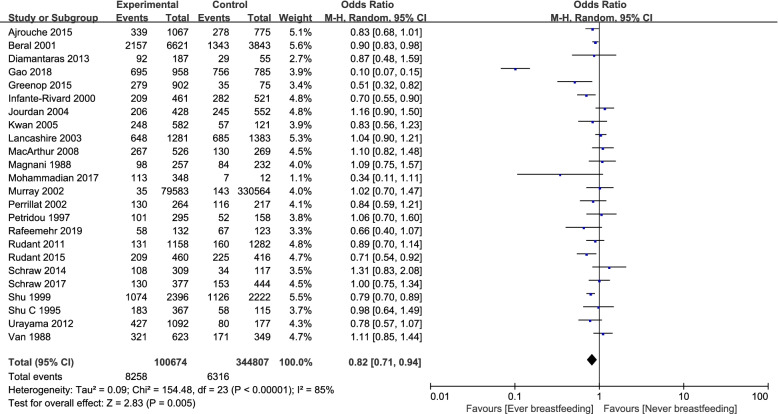
Forest plot demonstrating the incidence of haematological malignancies in childre

Whatever modes of breastfeeding and any length of breastfeeding were associated with lower risk of hematological malignancy (OR = 0.82, 95%CI, 0.71–0.94) (Fig. 5).

Separate subgroup analyses were also performed for leukemia (ALL, AML, and ANLL) and lymphoma (HL and NHL). For leukemia, there were 19 studies [8, 16–19, 29, 32, 36, 39, 40, 42, 43, 52–54, 56, 60, 62, 66] that indicated a statistically significant inverse relationship between any breastfeeding and leukemia risk (OR = 0.89, 95%CI, 0.82–0.97). Based on subgroup analysis of leukemia, the odds ratio for ALL was 0.85 (95%CI, 0.82–0.97), suggesting a protective effect of breastfeeding against ALL. Only three studies [19, 53, 62] were conducted containing subgroup analyses of AML (OR = 0.79, 95%CI, 0.66–0.94), and there was a significant relationship between AML and incidence of childhood cancer. For ANLL, three studies [16–18] were included, and there was no association between any breastfeeding and risk of ANLL (OR = 1.10, 95%CI, 0.68–1.80) **(**Table 1**)**.

The subgroup analysis for lymphoma included five studies [9, 16, 18–20] (OR = 0.93, 95%CI, 0.79–1.09). It found that breastfeeding had no significant protective effect on the risk of either HL [9, 18–20] or NHL [9, 16, 18–20] (OR = 0.89, 95%CI, 0.68–1.15; OR = 0.96, 95%CI, 0.79–1.17, respectively) **(**Table 1**)**.

#### Other childhood cancers

The data regarding the relationships of breastfeeding and other disease systems were also collected and analyzed. Nine studies [9, 34, 35, 38, 39, 49, 50, 57, 65] explored the association of breastfeeding and the incidence of nervous system diseases, and statistical significance was observed in subgroup analysis of relationship between breastfeeding and nervous system diseases (OR = 0.72, 95%CI, 0.54–0.96). However, there was investigated the effect breastfeeding on the incidence of cancers of skeletal [41], urinary [50], reproductive [65] and sensory systems [38]. No study mentioned the respiratory, digestive or endocrine systems. On the basis of the data we obtained, the results of incidence of childhood cancer in several other systems (skeletal: OR = 0.85, 95%CI, 0.62–1.16, reproductive: OR = 1.11, 95%CI, 0.73–1.67, sensory: OR = 0.77, 95%CI, 0.39–1.50) were not statistically significant; the exception was the urinary system (OR = 0.69, 95%CI, 0.51–0.92) (Table 1).

## Discussion

Childhood cancer is a major cause for death of children and adolescents in many countries. The relationship of breastfeeding and the incidence of childhood cancer had been addressed by several researchers. This meta-analysis and systematic review was conducted to explore the relationships between breastfeeding and childhood cancer, and a total of 46 studies were included in this study. To the best of our knowledge, there have been several meta-analyses and systematic reviews reporting these associations. We further investigated some other aspects of breastfeeding, involving modes and duration of breastfeeding and systemic cancers such as leukemia and cancers of the skeletal system, but it found that several meta-analyses and original studies had returned inconsistent results or suggestions regarding this topic. Mammas et al. [13] discussed the association between breastfeeding and viral infections, and stated that breastfeeding may help prevent infections during the first years of life. Two studies [11, 14] were more comprehensive than that of Mammas et al. Davis et al. [70], a review of nine published case-control published in 1998, drew the conclusion that breastfeeding for 6 months and beyond was more effective than short-term breastfeeding in terms of reducing the incidence of Hodgkin’s disease. However, there was no evidence to show an association between infant feeding and any other cancer. Rodent et al. [15], Amitay et al. [4], Darcy et al. [10] and Kwan et al. [12] found that there was an inverse correlation between any breastfeeding and childhood acute lymphoblastic leukemia, and that breastfeeding for 6 months and beyond was superior to short-term feeding in terms of reducing leukemia morbidity and mortality. They found no evidence to suggest that long-term breastfeeding was protective against AML.

The modes of breastfeeding were classified as exclusive or non-exclusive, and various modes of breastfeeding were subjected to subgroup analysis. In addition, we investigated the relative effects of three types, including differences between exclusive breastfeeding and non-exclusive breastfeeding, differences between mixed breastfeeding and non-mixed breastfeeding, and differences between bottle feeding and non-formula feeding, for cancer in children. The findings of subgroup analysis suggested that mixed and exclusive breastfeeding were superior to only bottle feeding and that the combination of two modes of breastfeeding was more beneficial than one alone. These findings suggested that the inclusion of breastfeeding might reduce the incidence of childhood cancer, regardless of the modes of breastfeeding. In other words, mixed-breastfeeding was better for children than exclusive bottle-feeding and exclusive breastfeeding.

There were some differences of results in this meta-analysis compared to the WHO suggestions regarding modes of breastfeeding. The latter recommended that exclusive breastfeeding was needed for the first 6 months of life and should be continued breastfeeding until 2 years of age or longer with the addition of appropriate supplements. However, no significant difference was found in this meta-analysis regarding the protective effect of exclusive breastfeeding and mixed feeding for 0–6 months, specifically for childhood cancer. The difference might derive from the small study populations due to there being only three studies [33, 39, 47] in this meta-analysis.

According to the results of duration of breastfeeding in this meta-analysis, there was protective effect for any duration of breastfeeding. However, there was no significant difference between ≥12 months and < 12 months of breastfeeding, suggesting that more than 6 months of breastfeeding could reduce the incidence of childhood cancer even more. Based on this dose-response relationship, it observed that, as the duration of breastfeeding increased, the odds of cancer significantly decreased. This suggested that extending the duration of breastfeeding as long as possible would reduce the incidence of cancer.

We removed the three largest and high-quality studies for separate analysis. These studies suggested weak significant inverse associations between any breastfeeding and long-term breastfeeding and childhood cancer, suggesting a very small influence of these studies on the results of this meta-analysis and systematic review.

Twenty-four studies [8, 16, 18–20, 29, 31–33, 36, 39, 40, 42, 43, 47, 52–56, 60, 62, 64, 66] reported hematological malignancies and were included in this meta-analysis. Common hematological malignancies, such as ALL, AML, ANLL, HL and NHL, were included in a subgroup analysis. Four meta-analyses [4, 12, 14, 15] probed the association between breastfeeding and the incidence of childhood leukemia. Based on subgroup analysis of leukemia, we found that breastfeeding indeed protected children by reducing the incidence of leukemia in childhood. This finding was consistent with conclusions of previous meta-analyses and systematic reviews. Undoubtedly, for ALL and AML, the functions of breastfeeding were identical because most leukemia cases were acute leukemia; nevertheless, there was no evidence to suggest that breastfeeding was associated with ANLL or lymphoma (HL and NHL).

The most comprehensive systemic diseases to date were analyzed in this meta-analysis and systematic review with respect to the relationship of breastfeeding with childhood cancer. According to the available evidence, breastfeeding reduced the prevalence of childhood hematological malignancies and cancers of the nervous and urinary systems. There were no remarkable associations of breastfeeding with cancers of the skeletal, reproductive, or sensory systems. Nevertheless, we cannot rule out that there may be variation because of the small sample size. Moreover, we cannot draw a conclusion regarding the relationship of breastfeeding with the incidence of cancers of other systems (i.e., respiratory, digestive, and endocrine systems) because of the absence of data.

The data from developed countries were considered separately from those of countries accounting for the majority of the collected data in this meta-analysis. Therefore, the conclusions needed to be confirmed further and compared with those of the total sample. The protective effect in the total sample was better than that of the developed countries. The hypothesis was that developing countries may do better than developed countries with respect to breastfeeding. The relationship of breastfeeding to childhood cancer in developed and developing countries needed to be further explored in the future.

There were several limitations of this meta-analysis. Firstly, the data collected from single studies were not complete and available, and some negative results might be missed, this might produce certain biases in the meta-analysis. In addition, unpublished literature was not included in terms of eligibility criteria. Secondly, the definitions of some studies did not clearly specify mixed breastfeeding, exclusive breastfeeding or bottle feeding, and the data were insufficient. Furthermore, the sample sizes of studies regarding the relationship between breastfeeding and cancers of certain systems of childhood were small, and this may affected the reliability of the meta-analysis. Thirdly, Most of populations included in this study come from high-income countries, and there was a lack of evidence support from low-income countries. Therefore, the conclusion of this study may only be applicable to population in high-income countries, and evidence applicable to population in low-income countries needs to be further produced and verified. In the course of cancer development, children may be exposed to more uncertain potential cancer risk factors. However, the original study did not provide relevant information about the potential cancer risk of children, which may have some effect on the accuracy of results. Finally, there was no information on covariates that may serve as mediators or confounders, including parental smoking behavior and family history of cancer.

## Conclusions

Current evidence demonstrated that breastfeeding have a potential protective role in preventing selective childhood cancer growth, especially in ALL and AML among hematological malignancies. However, breastfeeding did not appear to protect children from ANLL, HL, or NHL. Breastfeeding appeared to be beneficial in reducing the incidence of childhood cancer of the nervous and urinary systems. There was no evidence that breastfeeding was inversely related to the incidence of childhood malignancies of the skeletal, reproductive, or sensory systems. Notably, this meta-analysis indicated that the modes of mixed-breastfeeding and exclusive breastfeeding were recommended for preventing selective childhood cancer growth. This study recommended that breastfeeding be extended for as long as possible or maintained for at least 6 months to prevent selective childhood cancer growth. In addition, the current evidences were from population in high-income countries, and its applicability in low-income countries needed to be verified by evidences from population in low-income countries in the future.

## Supplementary Information

**Additional file 1: Table 1.** Main characteristics of the studies included in the meta-analysis. **Table 2.** The reasons of exclude literature. **Table 3.** Quality assessment of included studies in the meta-analysis using the Newcastle-Ottawa scale (NOS).

## Data Availability

All data generated or analysed during this study are included in this published article.

## Abbreviations

ALL: Acute lymphoblastic leukaemia
AML: Acute myeloid leukaemia
ANLL: Acute non-lymphoblastic leukaemia
CI: Confidence interval
HL: Hodgkin’s lymphoma
NHL: Non-Hodgkin’s lymphoma
NOS: Newcastle-Ottawa Scale
OR: Odds ratio
WHO: World Health Organization
