# Supplementary material for: Association between maternal breastfeeding and risk of systemic neoplasms of offspring
Source: Ital J Pediatr. 2022 Jun 16;48:98. doi: 10.1186/s13052-022-01292-9 (PMC9205047; doi:10.1186/s13052-022-01292-9)
Supplement: Supplementary file 1 — Additional file 1: Table 1. Main characteristics of the studies included in the meta-analysis. Table 2. The reasons of exclude literature. Table 3. Quality assessment of included studies in the meta-analysis using the Newcastle-Ottawa scale (NOS). [file 13052_2022_1292_MOESM1_ESM.docx]

**Legends of additional file 1**

**Additional file 1: Table S1** Main characteristics of the studies included in the meta-analysis

| **Study** | **Year** | **Country** | **Design** | **Cancer types** | **Mean±SD, Age Range of Cases** | **Gender (F/M)** | **No. of Cases/Controls** | **Method Assessing Infant Feeding** | **Exposure**  **(month)** | **Controls**  **(month)** | **Adjustment factors** |
| --- | --- | --- | --- | --- | --- | --- | --- | --- | --- | --- | --- |
| Rafieemehr | 2019 | *Iran* | Case-Control | ALL | 0-15Y | 52/73;  51/79 | (58,74)/(67,56) | Maternal interview(questionnaire) | Mix: 0-3,4-6,7-9,10-12,≥12 | Never | Age, gender, residence location |
| Hyland | 2018 | United States | Population based; Case-Control | Childhood Leukemia | 0-14Y | 114/137; 294/283 | (113,265)/(138,312) | Maternal interview(questionnaire) | Mix:<6 | Mix:≥6 | Child sex, birth year, and socioeconomic status |
| Gao | 2018 | China | Case-Control | Childhood leukemia | 0-14Y | 378/580; 336/449 | Exclusive:(489,529), Mix:(206,227), Formula:(263,29) | Medical records | Mix:0-3,4-6,7-9,10-12;exclusive; formula | Never; mix: >12 | Not mentioned |
| Schraw | 2017 | USA | Population based; Case-Control | ALL | 0-14Y; cases4.4±3.1; control4.4±3.3 | 80/91; 162/180 | (130,247)/(153,291) | Medical records | Mix:3.5±4.0 | Formula: 12.0 ± 4.0;solids: 8.0 ±4.0 | Ethnicity; Race; Sex; Maternal smoking during; Birth weight; Older siblings; Breast-feeding(per additional month);Age at introduction to solids |
| Bailey | 2017 | France | Population based; Case-Control | Brain tumors(Ependymomas57;Astrocytomas108;Embryonal Tumours190; Other Gliomas104) | 0-14Y | 210/300; 1432/1670 | (266,1465)/(202,1237) | Maternal telephone interview | Mix:<3, ≥3;<6, ≥6 | Never | Age, sex, study of originage, relevant confounders |
| Mohammadian | 2017 | Iran | Case-Control | Leukemia | 8Y; 11m-17Y | 51/69; 105/135 | Exclusive: (107,224)/(10,16) | Parents interview(questionnaire) | Mix:<1; 1-6; 7-12;13-19;20-24;>24; Exclusive:0;<1;1-3;4-6;≥7 | Never; None-Exclusive | Child sex; Age at the beginning of the study(year);Birth Order; Residence Place; Family monthly incomes; Maternal age in pregnancy; Neonatal jaundice; Allergy; Breastfeeding; Breastfeeding at Birth; Exclusive Breastfeeding |
| Rios | 2016 | France | Population based; Case-Control | Neuroblastoma | <15Y | 171/186;817/966( ESCALE: 88/86;429/520 ESTELLE: 83/100;388/446) | (151,947)/(118,642) | Maternal telephone interview | Mix:<3,3-5, ≥6 | Never | Age;Sex;Birth-order;Maternal age;Urban satus of the residence and study |
| Greenop | 2015 | Australia | Population based; Case-Control | Childhood ALL and CBT | 0-14Y | ALL:146/176;323/356 CBT:122/177; 352/381 | ALL:(279,623)/(35,40), CBT:(254,676)/(24,50) | Maternal interview(questionnaire) | Exclusive:<3;≥3,<6; ≥6; Mix:<3; ≥3,<6; ≥6 | Formula | ALL:age;date of cases ;date of questionnaire return for controls; sex;State;maternal education;maternal age at birth;birth order;, proportion of optimum birth weight; Never breast fed.CBT: Never breast fed; age; date of questionnaire return for controls,;sex; State; parental education; year of birth,;maternal age; ethnicity; NICU stay |
| Rudant | 2015 | France | Population based; Case-Control | ALL | <15Y | Not mentioned | (209,251)/(225,191) | Maternal telephone interview(questionnaire) | Mix | Never | Age and gender, and for non-genetic factors, parental socio-professional category. |
| Kucukcongar | 2015 | Turkey | Case-Control | Leukemia; lymphoma and solid tumors | 7.2±4.7Y, 6m-16Y | 114/186;119/197 | (275,312)/(25,4) | Pediatric Oncology and Pediatric Hematology Departments in Gazi University | Never | Mix:0-6;7-12;13-18;19-24;≥25 | Age |
| Heck | 2015 | USA, Canada | Case-Control | Retinoblastoma | bilateral cases:1.1±0.9Y; unilateral cases: 2.0±1.7Y; 0-14Y | Not mentioned | bilateral cases:(74, 120)/(4, 14), unilateral cases:(137, 120)/(28, 14) | Parents telephone interview(questionnaire) | Never; Mix: 1-6;7-11;≥12 | None or Mix: <1 | Age;mother's race;mother's educational attainment;household income; |
| Ajrouche | 2015 | France | National registry-based; Case-Control | ALL | <15Y | Not mentioned | (339,728)/(278,497) | Maternal telephone interview(questionnaire) | Mix: <6;≥6 | Never | Age, gender, parental professional category, maternal age, and degree of urbanisation |
| Schraw | 2014 | USA | Population based; Case-Control | ALL | 0-14Y | 67/75;137/147 | Exclusively breastfed: (16/36); Mixed feeding: (92/161); Exclusively formula-fed: (33/84) | Maternal interview(questionnaire) | Exclusive; Mix | Formula | Race, age at interview, sex, ethnicity, maternal smoking during pregnancy, and birth weight category |
| Lupo | 2014 | USA | Case-Control | Rhabdomyosarcoma | 7.6±5.3Y; 0-20Y | 107/215;107/215 | (163, 175)/(156, 142) | Parents telephone interview(questionnaire) | Mix:0;<6;6-12;≥12; | Never | Age, race, sex, household income, maternal and paternal education |
| Diamantaras | 2013 | Greece | Case-Control | Leukemia | 5-14Y | 48/73;48/73 | (92,95)/(29,26) | Guardian interview(questionnaire) | Mix | Never | Age, sex |
| Urayama | 2012 | USA | Case-Control | Acute Lymphoblastic Leukemia | <15Y | Non-Hispanic white:113/152;165/243 Hispanic:152/168;211/229 | Non-Hispanic white:(200,327)/(31,37);Hispanic:(227,338)/(49,60) | Pediatric hospitals; Cancer Registry; Center for Health Statistics(personal interview) | Mix | Never | Age, sex, maternal age, maternal education, and annual household income |
| Crouch | 2012 | UK | Population based; Case-Control | Acute Lymphoblastic Leukemia | 2-14Y | Not mentioned | (281, 492)/(361, 499) | Medical records; parental interview;and population census | Never or Mix <1 | Mix: >1 | Age, sex, region of residence |
| Waly | 2011 | Oman | Case-Control | Childhood Leukemia | case: 12.1±2.72Y control : 13.5±2.85Y | 40/30;40/30 | <6:(6,2);6-12:(10,6);12-24:(53,57);>24:(3,5) | Maternal interview(questionnaire) | Mix: <6 | Mix:6-12;12-24;>24 | Age gender |
| Rudant | 2011 | France | National registry-based; Case-Control | Childhood HL, NHL | HL:5-12Y;  NHL:2-12Y | HL:67/61;372/476NHL:48/116;581/731 | HL:(57, 393)/(70, 450); NHL:(74, 634)/(90, 672) | Maternal telephone interview(questionnaire) | Mix: <6;≥6 | Never | Age, gender, parental professional category, degree of urbanization, maternal age at child birth and housing, for contact with animals |
| Ortega | 2008 | Spain | Hospital based; Case-Control | Childhood cancer | 6.5; 0.5-16Y | 74/113; 76/111 | (122,144)/(65,43) | Family telephone interview | Exclusive: < 8 weeks;8–15 weeks;16–23 weeks;≥24weeks;At least 2 months; At least 4 months | Formula | Age |
| MacArthur | 2008 | Canada | Population based; Case-Control | Childhood Leukemia | 0-15Y | 196/203; 196/203 | Not mentioned | Parents interview | Exclusive:≤3;≤6;≤12;>12; Mix:0-3;4-6;7-12 | Formula | Age, gender, area |
| Bener | 2008 | United Arab Emirates | Hospital based; Case-Control | Leukemia and lymphomas(ALL, HL, NHL) | 5.44±3.29Y;<15Y | 62/107; 62/107 | ALL:0-6:(53,169)/(26,169), >6:(50,169)/(72,169);HL:0-6:(17,169)/(8,169),>6:(15,169)/(23,169); NHL:0-6:(9,169)/(8,169), >6:(25,169)/(32,169) | Maternal telephone interview | Mix: 0-6 | Mix: >6 | Age, sex |
| Harding | 2007 | UK | Population based; Case-Control | Childhood CNS tumors | <15Y | Not mentioned | (402,4460)/(231,2495) | Maternal interview(questionnaire) | Mix: <1;1-6;>6 | Never | Age, sex, region, deprivation index |
| Altinkaynak | 2006 | Turkey | Population based; Case-Control | Childhood Acute Leukemia and Lymphomas | Leukemia:8.1 ±5.3Y;Malignant lymphoma: 9.5 ± 6.0Y 1-16Y | 51/86; 59/87 | ALL:(27,15)/(45,61), AML:(12,6)/(3,10), HL:(6,3)/(6,10), NHL:(12,8)/(26,33) | Maternal interview | Exclusive: 0-6; 0-≤1, 1-≤3;1-≤6 | Exclusive: >6 | Not mentioned |
| Saddlemire | 2006 | USA | Population based; Case-Control | Wilms tumor | <16Y | Not mentioned | Exclusive:(67,77), Mix:(243,255) | Maternal telephone interview | Exclusive, Mix: 0-3;4-6;7-9;10-12;>13 | Never | Child's age at reference date; geographic region of residence; household income; mother’s education |
| Kwan | 2005 | USA | Population based; Case-Control | Childhood acute lymphoblastic leukemia | 5,5±3.3Y;0-14Y | 147/164; 192/208 | (248,334)/(57,64) | Maternal interview(questionnaire) | Mix:≤3;4-6;7-12; ≥13 | Formula | Birth, sex, Hispanic ethnic status, maternal race and maternal county of residence at birth (only Phase one), annual household income, maternal education |
| Jourdan | 2004 | France | Population based; Case-Control | Acute leukemia(ALL, AML) | 0-15Y | 213/260;241/326 | ALL:(176,222)/(216,30),AML:(30,222)/(29,307) | Maternal interview(questionnaire) | Mix: <3;3-6;>6 | Never | Gender, age at diagnosis, region of residence at diagnosis |
| Lancashire | 2003 | UK | Case-Control | All childhood cancers(ALL, Other leukemia; Other reticuloendothelial cancers, All other cancers | 0-16Y | Not mentioned | ALL:(461,452)/(480,490), Other leukemia: (187,181)/(205,208), Other reticuloendothelial cancers:(163,142)/(160,182),All other cancers: (777,797)/(901,901) | Parents interview(questionnaire) | Mix: <1;1-6; ≥7 | Never | Sex, age at death, father's of Case-Control upational social class, sibship position, maternal age at child's birth |
| Perrillat | 2002 | France | Hospital based; Case-Control | Childhood acute leukemia(ALL, ANLL) | 0-15Y | Not mentioned | ALL: (114,134)/(104,101), ANLL: (16,134)/(12,101) | Maternal interview | Mix:<3;3-5;6-11;≥12 | Never | Age, gender,hospital, ethnic origin,maternal educational level,parental socioprofessional category, number of pregnancies, birth weight, duration of pregnancy |
| Perrillat | 2002 | France | Case-Control | Childhood acute leukemia | 0-15Y | 115/166; 122/168 | (130,134)/(116,101) | Maternal interview | Mix: <6; ≥6 | Never | Age, gender, hospital, ethnic origin, maternal educational level, parental socio professional category, birth weight, length of pregnancy, number of previous pregnancies |
| Murray | 2002 | UK | Case-Control | ALL | 0-16Y | 87/101;210785/223945 | (35,79548)/(143,330421) | Maternal interview | Mix | Never | Sex |
| Schuz | 2001 | German | Population based; Case-Control | Pediatric tumors(Astrocytoma Medulloblastoma Ependymoma) | <15Y | 204/262;1066/1392(Astrocytoma:60/59 Medulloblastoma :37/75Ependymoma:26/24) | Astrocytomas: (70,2438)/(49,960), Ependymomas: (34,2438)/(16,960), Medulloblastomas: (71,2438)/(41,960) | Parents telephone interview(questionnaire) | Mix: 2-6, >6 | Mix: ≤1 | Gender, age groups of 1 year, and year of  birth , degree of urbanization and socioeconomic status. |
| Hardell | 2001 | Sweden | Case-Control | Childhood cancer | 0-14Y | 403/432; 422/438 | All:(716,744)/(119,116), Hodgkin’s disease: (21,24)/(7,6), Brain tumours: (223,242)/(41,32), Neuroblastoma,ganglioneuroblastoma: (28,34)/(6,4), Renal tumour: (44,49)/(7,4), Liver cancer: (12,15)/(4,1), Germ cell,trophoblastic,other gonadal neoplasms: (18,21)/(3,2) | Medical records | Mix: ≥1(1-<6; ≥6) | Mix: <1 | Age, sex |
| Bener | 2001 | United Arab Emirates | Case-Control | Leukemia and Lymphomas (ALL, HL, NHL) | 2-14Y | Not mentioned | ALL:(37,22)/(32,47), HL:(10,4)/(12,18), NHL:( 9,3 )/(17,23) | Maternal telephone interview | Exclusive: 0-6 | Exclusive: >6 | Age, sex, ethnicity |
| Beral | 2001 | UK | Population based; Case-Control | Childhood cancer(leukemia(HL, NHL, lymphoma and other cancer )) | 0-14Y | Not mentioned | Leukaemia: 1008/629, Hodgkin's  disease: 71/43, NHL: 149/79, Other cancer: 929/592 | Maternal interview(questionnaire) | Mix: <1;1-6;≥7 | Formula | Age at diagnosis, sex, region, birth order and deprivation index |
| Infante | 2000 | Canada | Population based; Case-Control | ALL | 0-9Y | Not mentioned | (209,252)/(282,239) | Maternal telephone interview | Mix: ≤3;>3 | Never | Maternal age, level of schooling |
| Smulevich | 1999 | Moscow | Population based | All sites(Leukemia Hodgkin’s disease Non-Hodgkin’s lymphoma Soft-tissue sarcoma Neuroblastoma Nephroblastoma Brain and spinal-cord cancer) | 0-14Y | Not mentioned | <1(51,17), 1-2(54,138), 3-4(166,315), 5-6(103,184), 7-12(146,358), >12(73,169) | Parents interview | Mix: <1;1-2; 3-4; 5-6; 7-12 | Mix: >12 | Age, gender and residence |
| Shu | 1999 | United States, Canada, and Australia | Population based; Case-Control | Childhood Acute Leukemia(AML, ALL) | ALL:0-15Y  AML:0-18Y | Not mentioned | AML:(190,266)/(266,273), ALL:(884,1056)/(860,823) | Maternal telephone interview | Mix:≤6;>6;1-3;4-6;7-9;10-12;>12 | Never | Maternal race, maternal education, and family annual income |
| Schuz | 1999 | German | Case-Control | Childhood leukemia | 0-14Y | Leukaemias:412/598; 412/598;ALL:292/394;1084/1504 | Leukemia:(1001,1001), ALL:(682,2574) | Telephone interview(questionnaire) | Mix: ≤1;2-6 | Mix: >6 | Gender,age, year of birth, study setting, SES, degree of urbanization |
| Petridou | 1997 | Greece | Hospital based; Case-Control | Childhood leukemia | 0-14Y | 68/85;132/168 | (101,194)/(52,106) | Interviewer-administered questionnaire | Mix | Never | Sex, age, residence, sociodemographic, lifestyle, environmental and biomedical variables |
| Shu | 1995 | US,Canada | Case-Control | MGCT(malignant germ-cell tumor) | 0-15Y | 67/38;308/331 | (58,337)/(47,302) | Self-administered(questionnaire) | Mix:1-6;7-12;>12 | Never | Age,gender,gestational age,number of livebirths,maternal education,smoking during pregnancy |
| Shu | 1995 | China | Population based; Case-Control | Childhood Lymphoma and Leukemia | <15Y | Not mentioned | Lymphoma: ALL: (62,68)/(20,14), HL:(11,12)/(3,2), NHL(51,56)/(17, 12);  Acute leukaemia: ALL:(121,116)/(38,43),Acute leukaemia: (121,116)/(38,43), ALL:(82,79)/(26,29), ANLL(39,37)/(12,14) | Parents interview(questionnaire) | Mix: 1-6;>6 | Never | Sex, maternal age at birth, birthweight, maternal working status, Case-Controlupational exposure to chemicals during infancy. |
| Magnani | 1988 | Italy | Case-Control | ALL, ANLL, NHL | Not mentioned | Not mentioned | ALL:76/65, ANLL:12/10, NHL:10/9 | Parents interview | Mix: 1-6; ≥7 | Never | Maternal age at the child’s birth, socioeconomic status |
| Davis | 1988 | USA | Case-Control | Childhood cancer(ALL, other Leukaemias, Brain, STS, Lymphoma, other cancers(Hodgkin's, NHL)) | 1.5-15Y | 81/120;73/108 HL:5/8;73/108 NHL:5/8;73/108 | ALL:52, other Leukaemias: 11, Brain: 38, STS: 15, Lymphoma: 26, other cancers: 59, Hodgkin's:(5,107)/(8,74),NHL:(8,107)/(5,74) | Medical records | Mix: ≤6, >6 | Never | Age, sex, area of residence |
| Hartley | 1988 | UK | Case-Control | Childhood cancer | <15Y | Not mentioned | Prectitioner control:245/281, Hospital control:219/305 | Parents interview(questionnaire) | Mix | Never | Age, sex |
| Van | 1988 | Netherlands | Population based;Case-Control | ALL | <15Y | Not mentioned | (321, 302)/(171, 178) | Parents interview(questionnaire) | Mix: ≤6;>6 | Formula | Age, sex, birth order, social class, maternal education, smoking,alcohol use of mother during pregnance |

**Note:** M, month; Y, Year; ALL, Acute lymphocytic leukemia; AML, Acute myeloid leukemia; ANLL, Acute non-lymphocytic leukemia; HL, Hodgkin's lymphoma; NHL, Non-Hodgkin's lymphoma.

**References**

Bray F, Ferlay J, Soerjomataram I, et al. Global cancer statistics 2018: GLOBOCAN estimates of incidence and mortality worldwide for 36 cancers in 185 countries. CA Cancer J Clin 2018;68:394-424.

Kaatsch P. Epidemiology of childhood cancer. Cancer Treat Rev 2010;36:277-85.

Li J, Thompson TD, Miller JW, et al. Cancer incidence among children and adolescents in the United States, 2001-2003. Pediatrics 2008;121:e1470-7.

Eidelman AI. Breastfeeding and the use of human milk: an analysis of the American Academy of Pediatrics 2012 Breastfeeding Policy Statement. Breastfeed Med 2012;7:323-4.

Wright CM, Cox KM, Le Couteur A. How does infant behaviour relate to weight gain and adiposity? Proc Nutr Soc 2011;70:485-93.

Horta B, Victora CG. Long-term eﬀects of breastfeeding: a systematic review. World Health Organization 2013:69.

H BG, Bellamy C. Global Strategy for Infant and Young Child Feeding. World Health Organization 2003:30.

Cota GF, de Sousa MR, Fereguetti TO, et al. Efficacy of anti-leishmania therapy in visceral leishmaniasis among HIV infected patients: a systematic review with indirect comparison. PLoS Negl Trop Dis 2013;7:e2195.

Schraw JM, Scheurer ME, Forman MR. A Vulnerable Age for the Introduction of Solid Foods in Pediatric Acute Lymphoblastic Leukemia. Nutr Cancer 2017;69:261-6.

Bailey HD, Rios P, Lacour B, et al. Factors related to pregnancy and birth and the risk of childhood brain tumours: The ESTELLE and ESCALE studies (SFCE, France). Int J Cancer 2017;140:1757-69.

Mohammadian M, Moghaddam AA, Mahdavifar N. Investigating the relationship between breastfeeding with childhood leukemia in sistan and baluchestan province. Iranian Journal of Blood and Cancer 2017;9:89.

Amitay EL, Dubnov Raz G, Keinan-Boker L. Breastfeeding, Other Early Life Exposures and Childhood Leukemia and Lymphoma. Nutr Cancer 2016;68:968-77.

Rios P, Bailey HD, Orsi L, et al. Risk of neuroblastoma, birth-related characteristics, congenital malformations and perinatal exposures: A pooled analysis of the ESCALE and ESTELLE French studies (SFCE). Int J Cancer 2016;139:1936-48.

Greenop KR, Bailey HD, Miller M, et al. Breastfeeding and nutrition to 2 years of age and risk of childhood acute lymphoblastic leukemia and brain tumors. Nutr Cancer 2015;67:431-41.

Rudant J, Orsi L, Bonaventure A, et al. ARID5B, IKZF1 and non-genetic factors in the etiology of childhood acute lymphoblastic leukemia: the ESCALE study. PLoS One 2015;10:e0121348.

Kucukcongar A, Oguz A, Pinarli FG, et al. Breastfeeding and Childhood Cancer: Is Breastfeeding Preventative to Childhood Cancer? Pediatr Hematol Oncol 2015;32:374-81.

Heck JE, Omidakhsh N, Azary S, et al. A case-control study of sporadic retinoblastoma in relation to maternal health conditions and reproductive factors: a report from the Children's Oncology group. BMC Cancer 2015;15:735.

Ajrouche R, Rudant J, Orsi L, et al. Childhood acute lymphoblastic leukaemia and indicators of early immune stimulation: the Estelle study (SFCE). Br J Cancer 2015;112:1017-26.

Schraw JM, Dong YQ, Okcu MF, et al. Do longer formula feeding and later introduction of solids increase risk for pediatric acute lymphoblastic leukemia? Cancer Causes Control 2014;25:73-80.

Lupo PJ, Zhou R, Skapek SX, et al. Allergies, atopy, immune-related factors and childhood rhabdomyosarcoma: a report from the Children's Oncology Group. Int J Cancer 2014;134:431-6.

Diamantaras AA, Dessypris N, Sergentanis TN, et al. Nutrition in early life and risk of childhood leukemia: a case-control study in Greece. Cancer Causes Control 2013;24:117-24.

Urayama KY, Chokkalingam AP, Metayer C, et al. HLA-DP genetic variation, proxies for early life immune modulation and childhood acute lymphoblastic leukemia risk. Blood 2012;120:3039-47.

Crouch S, Lightfoot T, Simpson J, et al. Infectious illness in children subsequently diagnosed with acute lymphoblastic leukemia: modeling the trends from birth to diagnosis. Am J Epidemiol 2012;176:402-8.

Waly MI, Ali A, Al-Saadoon M, et al. Breastfeeding is not associated with risk of developing childhood leukemia in the Sultanate of Oman. Asian Pac J Cancer Prev 2011;12:2087-91.

Rudant J, Orsi L, Monnereau A, et al. Childhood Hodgkin's lymphoma, non-Hodgkin's lymphoma and factors related to the immune system: the Escale Study (SFCE). Int J Cancer 2011;129:2236-47.

Flores-Lujano J, Perez-Saldivar ML, Fuentes-Panana EM, et al. Breastfeeding and early infection in the aetiology of childhood leukaemia in Down syndrome. Br J Cancer 2009;101:860-4.

Ortega-Garcia JA, Ferris-Tortajada J, Torres-Cantero AM, et al. Full breastfeeding and paediatric cancer. J Paediatr Child Health 2008;44:10-3.

MacArthur AC, McBride ML, Spinelli JJ, et al. Risk of childhood leukemia associated with vaccination, infection, and medication use in childhood: the Cross-Canada Childhood Leukemia Study. Am J Epidemiol 2008;167:598-606.

Bener A, Hoffmann GF, Afify Z, et al. Does prolonged breastfeeding reduce the risk for childhood leukemia and lymphomas? Minerva Pediatr 2008;60:155-61.

Harding NJ, Birch JM, Hepworth SJ, et al. Breastfeeding and risk of childhood CNS tumours. Br J Cancer 2007;96:815-7.

Saddlemire S, Olshan AF, Daniels JL, et al. Breast-feeding and Wilms tumor: a report from the Children's Oncology Group. Cancer Causes Control 2006;17:687-93.

Altinkaynak S, Selimoglu MA, Turgut A, et al. Breast-feeding duration and childhood acute leukemia and lymphomas in a sample of Turkish children. J Pediatr Gastroenterol Nutr 2006;42:568-72.

Kwan ML, Buffler PA, Wiemels JL, et al. Breastfeeding patterns and risk of childhood acute lymphoblastic leukaemia. Br J Cancer 2005;93:379-84.

Jourdan-Da Silva N, Perel Y, Mechinaud F, et al. Infectious diseases in the first year of life, perinatal characteristics and childhood acute leukaemia. Br J Cancer 2004;90:139-45.

Lancashire RJ, Sorahan T. Breastfeeding and childhood cancer risks: OSCC data. Br J Cancer 2003;88:1035-7.

Perrillat F, Clavel J, Jaussent I, et al. Breast-feeding, fetal loss and childhood acute leukaemia. Eur J Pediatr 2002;161:235-7.

Perrillat F, Clavel J, Auclerc MF, et al. Day-care, early common infections and childhood acute leukaemia: a multicentre French case-control study. Br J Cancer 2002;86:1064-9.

Murray L, McCarron P, Bailie K, et al. Association of early life factors and acute lymphoblastic leukaemia in childhood: historical cohort study. Br J Cancer 2002;86:356-61.

Schuz J, Kaletsch U, Kaatsch P, et al. Risk factors for pediatric tumors of the central nervous system: results from a German population-based case-control study. Med Pediatr Oncol 2001;36:274-82.

Hardell L, Dreifaldt AC. Breast-feeding duration and the risk of malignant diseases in childhood in Sweden. Eur J Clin Nutr 2001;55:179-85.

Bener A, Denic S, Galadari S. Longer breast-feeding and protection against childhood leukaemia and lymphomas. Eur J Cancer 2001;37:234-8.

Infante-Rivard C, Fortier I, Olson E. Markers of infection, breast-feeding and childhood acute lymphoblastic leukaemia. Br J Cancer 2000;83:1559-64.

Smulevich VB, Solionova LG, Belyakova SV. Parental occupation and other factors and cancer risk in children: I. Study methodology and non-occupational factors. Int J Cancer 1999;83:712-7.

Shu XO, Linet MS, Steinbuch M, et al. Breast-feeding and risk of childhood acute leukemia. J Natl Cancer Inst 1999;91:1765-72.

Schuz J, Kaletsch U, Meinert R, et al. Association of childhood leukaemia with factors related to the immune system. Br J Cancer 1999;80:585-90.

Petridou E, Trichopoulos D, Kalapothaki V, et al. The risk profile of childhood leukaemia in Greece: a nationwide case-control study. Br J Cancer 1997;76:1241-7.

Shu XO, Nesbit ME, Buckley JD, et al. An exploratory analysis of risk factors for childhood malignant germ-cell tumors: report from the Childrens Cancer Group (Canada, United States). Cancer Causes Control 1995;6:187-98.

Shu XO, Clemens J, Zheng W, et al. Infant breastfeeding and the risk of childhood lymphoma and leukaemia. Int J Epidemiol 1995;24:27-32.

van Duijn CM, van Steensel-Moll HA, van der Does-vd Berg A, et al. Infant feeding and childhood cancer. Lancet 1988;2:796-7.

Magnani C, Pastore G, Terracini B. Infant feeding and childhood cancer. Lancet 1988;2:1136.

Hartley AL, Birch JM, McKinney PA, et al. The Inter-Regional Epidemiological Study of Childhood Cancer (IRESCC): past medical history in children with cancer. J Epidemiol Community Health 1988;42:235-42.

Davis MK, Savitz DA, Graubard BI. Infant feeding and childhood cancer. Lancet 1988;2:365-8.

Hyland C, Gunier RB, Metayer C, et al. Maternal residential pesticide use and risk of childhood leukemia in Costa Rica. Int J Cancer 2018;143:1295-304.

Gao Z, Wang R, Qin ZX, et al. Protective effect of breastfeeding against childhood leukemia in Zhejiang Province, P. R. China: a retrospective case-control study. Libyan J Med 2018;13:1508273.

Investigators UCCS. Breastfeeding and childhood cancer. Br J Cancer 2001;85:1685-94.

**Additional file 1: Table S2** The reasons of exclude literature

| **Study** | **Year** | **Title** | **Reasons** |
| --- | --- | --- | --- |
| Dodge, J. A | 1975 | Infantile hypertrophic pyloric stenosis in Belfast, 1957-1969 | The outcome did not meet inclusion criteria |
| Jelliffe, D. B | 1979 | Human milk and breast feeding: recent highlights | Review |
| Schwerin, H. S | 1981 | Food eating patterns and health: a reexamination of the Ten-State and HANES I surveys | Study design did not meet inclusion criteria |
| Smigel, K. L | 1988 | Breast-feeding linked to decreased cancer risk for mother, child | Review |
| Mori, M. | 1988 | A case-control study of testicular cancer | Study design did not meet inclusion criteria |
| Kapil, U | 1989 | Knowledge and attitude among child development project officers towards breast feeding | The exposure did not match |
| Mori, M | 1990 | Maternal factors of testicular cancer: a case-control study in Japan | The exposure did not match |
| Jeannel, D | 1990 | Diet, living conditions and nasopharyngeal carcinoma in Tunisia--a case-control study | The exposure did not match |
| Birch, J. M | 1990 | The inter-regional epidemiological study of childhood cancer (IRESCC): case-control study of children with central nervous system tumours | The exposure did not match |
| Schwartzbaum, J. A | 1991 | An exploratory study of environmental and medical factors potentially related to childhood cancer | Study design did not meet inclusion criteria |
| Forman, M | 1992 | Factors influencing milk insufficiency and its long-term health effects: the Bedouin Infant Feeding Study | The outcome did not meet inclusion criteria |
| No authors listed | 1994 | Is breast feeding beneficial in the UK? Statement of the standing Committee on Nutrition of the British Paediatric Association | The outcome did not meet inclusion criteria |
| Donnelly, B. W | 1995 | The pediatrician and cancer prevention | Study design did not meet inclusion criteria |
| Hamre, M | 1997 | Langerhans cell histiocytosis: an exploratory epidemiologic study of 177 cases | Review |
| Kusuhara, K | 1997 | Breast milk is not a significant source for early Epstein-Barr virus or human herpesvirus 6 infection in infants: a seroepidemiologic study in 2 endemic areas of human T-cell lymphotropic virus type I in Japan | The exposure did not match |
| Wilkins, J. R. | 1997 | Comparing dietary recall data for mothers and children obtained on two occasions in a case-control study of environmental factors and childhood brain tumours | The exposure did not match |
| Villalpando, S | 1998 | Early and late effects of breast-feeding: does breast-feeding really matter? | Review |
| Udall, J | 1999 | Cow's milk versus formula in older infants: consequences for human nutrition | The exposure did not match |
| Fornarini, B | 1999 | Human milk 90K (Mac-2 BP): possible protective effects against acute respiratory infections | The outcome did not meet inclusion criteria |
| Davis, M. K | 2001 | Breastfeeding and chronic disease in childhood and adolescence | Review |
| Daniels, J. L | 2002 | Breast-feeding and neuroblastoma, USA and Canada | Study design did not meet inclusion criteria |
| Hrusak, O | 2002 | Acute lymphoblastic leukemia incidence during socioeconomic transition: selective increase in children from 1 to 4 years | The exposure did not match |
| Uzcudun, A. E | 2002 | Nutrition and pharyngeal cancer: results from a case-control study in Spain | The exposure did not match |
| Cerhan, J. R | 2002 | Menstrual and reproductive factors and risk of non-Hodgkin lymphoma: the Iowa women's health study (United States) | The participants did not meet inclusion criteria |
| Fear, N. T | 2003 | Re: Breast-feeding and neuroblastoma, USA and Canada | Only abstract |
| Corley, R. A | 2003 | Evaluation of physiologically based models of pregnancy and lactation for their application in children's health risk assessments | Study design did not meet inclusion criteria |
| Barnard, N. D | 2003 | The milk debate goes on and on and on! | Study design did not meet inclusion criteria |
| No authors listed | 2003 | Position of the American Dietetic Association and Dietitians of Canada: vegetarian diets | Study design did not meet inclusion criteria |
| Dundaroz, R | 2003 | Analysis of DNA damage using the comet assay in infants fed cow's milk | The outcome did not meet inclusion criteria |
| MacDonald, A | 2003 | Is breast best? Is early solid feeding harmful? | The outcome did not meet inclusion criteria |
| Kwan, M | 2004 | Breastfeeding and the risk of childhood leukemia: a meta-analysis | Mate analysis |
| McNally, R | 2004 | An infectious aetiology for childhood acute leukaemia: a review of the evidence | Review |
| Tripathy, A. K | 2004 | Breast Feeding and Childhood Hematological Malignancy | Review |
| Frentzel-Beyme, R | 2004 | Factors affecting the incident juvenile bone tumorsin an Austrian case–control study | The exposure did not match |
| Chen, A | 2004 | Breastfeeding and the risk of postneonatal death in the United States | The outcome did not meet inclusion criteria |
| Martin, R. M | 2005 | Breast-feeding and childhood cancer: A systematic review with metaanalysis | Review |
| Wang, R | 2005 | Human milk research for answering questions about human health | Review |
| Schack-Nielsen, L | 2005 | Long term effects of breastfeeding on the infant and mother | Review |
| Xu, H | 2005 | Induction of cytochrome P450 1A by cow milk-based formula: a comparative study between human milk and formula | The exposure did not match |
| Pearce, M. S | 2005 | Does increased duration of exclusive breastfeeding protect against Helicobacter pylori Infection? The Newcastle Thousand Families Cohort Study at age 49-51 years | The exposure did not match |
| Guise, J | 2005 | Review of case-control studies related to breastfeeding and reduced risk of childhood leukemia | The exposure did not match |
| van den Hazel, P | 2006 | Today's epidemics in children: possible relations to environmental pollution and suggested preventive measures | Review |
| Schack-Nielsen, L | 2006 | Breast feeding and future health | Review |
| Ergun, M. A | 2006 | Investigating the in vitro effect of taurine on the infant lymphocytes by sister chromatid exchange | Study design did not meet inclusion criteria |
| McNally, R | 2006 | Environmental factors and childhood acute leukemias and lymphomas | The exposure did not match |
| Rogers, I | 2006 | Milk as a food for growth? The insulin-like growth factors link | The exposure did not match |
| McNally, R. J | 2006 | Environmental factors and childhood acute leukemias and lymphomas | The exposure did not match |
| Franke, A. A | 2006 | Isoflavones in breastfed infants after mothers consume soy | The outcome did not meet inclusion criteria |
| McGlynn, K. A | 2006 | Maternal smoking and testicular germ cell tumors | The participants did not meet inclusion criteria |
| Turck, D | 2007 | Later effects of breastfeeding practice: the evidence | Review |
| Loland, B. F | 2007 | Human milk, immune responses and health effects | Study design did not meet inclusion criteria |
| O'Connor, S. M. | 2007 | Infectious etiologies of childhood leukemia: plausibility and challenges to proof | The exposure did not match |
| van der Pols, J | 2007 | Childhood dairy intake and adult cancer risk: 65-y follow-up of the Boyd Orr cohort | The participants did not meet inclusion criteria |
| Cattaneo, A. | 2008 | The benefits of breastfeeding or the harm of formula feeding? | Study design did not meet inclusion criteria |
| Sellers, T. A | 2008 | Unpasteurized milk consumption and subsequent risk of cancer | The participants did not meet inclusion criteria |
| James, D. C | 2009 | Position of the American Dietetic Association: promoting and supporting breastfeeding | Review |
| Ip, S | 2009 | A summary of the Agency for Healthcare Research and Quality's evidence report on breastfeeding in developed countries | Review |
| Zur Hausen, H | 2009 | Childhood leukemias and other hematopoietic malignancies: interdependence between an infectious event and chromosomal modifications | The exposure did not match |
| Melnik, B. C | 2009 | Milk--the promoter of chronic Western diseases | The exposure did not match |
| Karaolis-Danckert, N | 2009 | Birth and early life influences on the timing of puberty onset: results from the DONALD (DOrtmund Nutritional and Anthropometric Longitudinally Designed) Study | The outcome did not meet inclusion criteria |
| Kuitunen, M | 2009 | Pro- and prebiotic supplementation induces a transient reduction in hemoglobin concentration in infants | The outcome did not meet inclusion criteria |
| Flores-Lujano | 2009 | Breastfeeding and early infection in the aetiology of childhood leukaemia in Down syndrome | The participants did not meet inclusion criteria |
| Lucas, A | 2010 | Growth and later health: a general perspective | The outcome did not meet inclusion criteria |
| Ruder, E. H | 2011 | Adolescent and mid-life diet: risk of colorectal cancer in the NIH-AARP Diet and Health Study | Study design did not meet inclusion criteria |
| Blissett, J | 2011 | Relationships between parenting style, feeding style and feeding practices and fruit and vegetable consumption in early childhood | Study design did not meet inclusion criteria |
| Ogg, S. W | 2011 | Protective effects of breastfeeding for mothers surviving childhood cancer | The outcome did not meet inclusion criteria |
| Mosby, T. T | 2012 | Nutrition in adult and childhood cancer: role of carcinogens and anti-carcinogens | Study design did not meet inclusion criteria |
| Chung, C. S | 2012 | FDA's health claim review: whey-protein partially hydrolyzed infant formula and atopic dermatitis | The outcome did not meet inclusion criteria |
| O'Rorke, M. A | 2013 | Do perinatal and early life exposures influence the risk of malignant melanoma? A Northern Ireland birth cohort analysis | The participants did not meet inclusion criteria |
| Bauters, T | 2014 | Breastfeeding a child on treatment for childhood cancer | Review |
| Feltbower, R.G | 2014 | UK case control study of brain tumours in children, teenagers and young adults: a pilot study | The participants did not meet inclusion criteria |
| Amitay, E. L | 2015 | Breastfeeding and other early life exposures and the risk of childhood leukemia and lymphoma in Israel: A case control study | Letter |
| Gammill, H. S | 2015 | Breastfeeding and childhood leukemia incidence: duplicate data inadvertently included in the meta-analysis and consideration of possible confounders | Letter |
| Amitay, E. L | 2015 | Breastfeeding and Childhood Leukemia Incidence--Reply | Reply |
| Rudant, J | 2015 | Childhood acute lymphoblastic leukemia and indicators of early immune stimulation: a Childhood Leukemia International Consortium study | Review |
| Gunther, A. L | 2015 | Early diet and later cancer risk: prospective associations of dietary patterns during critical periods of childhood with the gh-igf axis, insulin resistance and body fatness in younger adulthood | Study design did not meet inclusion criteria |
| Mallory, J | 2016 | Breastfeeding during autologous stem cell transplant for medulloblastoma: A case report | Only abstract |
| Kannan Kutty, P | 2016 | Breastfeeding counsel against cancers | Review |
| Michie, C. | 2016 | Breast feeding could reduce the risk of childhood leukaemias | Review |
| Thorning, T. K | 2016 | Milk and dairy products: good or bad for human health? An assessment of the totality of scientific evidence | Study design did not meet inclusion criteria |
| Ekwueme, D | 2016 | Estimating health benefits and lifetime economic cost-savings from promoting breastfeeding to prevent childhood leukemia in the United States | Study design did not meet inclusion criteria |
| No authors listed | 2016 | Breastfeeding: achieving the new normal | Study design did not meet inclusion criteria |
| Whitehead, T. P | 2016 | Childhood leukemia and primary prevention | The exposure did not match |
| Paredes, G. G | 2016 | Neonatal cancer in peru: 18 years of experience in the rebagliati hospital | The outcome did not meet inclusion criteria |
| Ekwueme, D | 2016 | Estimating health benefits and lifetime economic cost-savings from promoting breastfeeding to prevent childhood leukemia in the United States | The outcome did not meet inclusion criteria |
| Amitay | 2016 | Breastfeeding, Other Early Life Exposures and Childhood Leukemia and Lymphoma | The participants did not meet inclusion criteria |
| Roess, A | 2017 | Trends in breastfeeding and cesarian sections over a 20 year period in 40 low and middle income countries | The outcome did not meet inclusion criteria |
| Mohammadian, M | 2017 | Investigating the relationship between breastfeeding with childhood leukemia in sistan and baluchestan province | The outcome did not meet inclusion criteria |
| Mammas, I | 2018 | Paediatric Virology and its interaction between basic science and clinical practice | Review |
| Kyriakopoulou | 2018 | Parental Occupational Exposures and Risk of Childhood Acute Leukemia | The exposure did not match |
| Perez-Sato | 2019 | Cow's Milk Consumption and Health: A Health Professional's Guide | Guideline |
| Gungor | 2019 | Infant milk-feeding practices and childhood leukemia: a systematic review | Review |
| Bailey | 2019 | The 2016 Feeding Infants and Toddlers Study (FITS): Dietary Intakes and Practices of Children in the United States from Birth to 48 Months | Study design did not meet inclusion criteria |
| Lauren M | 2019 | Metabolomics of neonatal blood spots reveal distinct phenotypes of pediatric acute lymphoblastic leukemia and potential effects of early-life nutrition | Study design did not meet inclusion criteria |
| Timms | 2019 | Exploring a potential mechanistic role of DNA methylation in the relationship between in uteroand post-natal environmental exposures and risk of childhood acute lymphoblastic leukaemia | The exposure did not match |
| Costas | 2019 | Reproductive Factors, Exogenous Hormone Use, and Risk of B-Cell Non-Hodgkin Lymphoma in a Cohort of Women From the European Prospective Investigation Into Cancer and Nutrition | The exposure did not match |

**Additional file 1: Table S3** Quality assessment of included studies in the meta-analysis using the Newcastle-Ottawa scale (NOS)

| **Source** | **Case Definition** | **Representativeness of the Cases** | **Selection of Controls** | **Definition of Controls** | **Comparability of Cases and Controls** | **Ascertainment of Exposure** | **Same Method of Ascertainment for Cases and Controls** | **Nonresponse Rate** | **Total NOS Score** |
| --- | --- | --- | --- | --- | --- | --- | --- | --- | --- |
| Rafieemehr et al,2019 | * | * | * | * | ** | * | * |  | 8 |
| Hyland et al,2018 | * | * | * | * | * | * | * |  | 7 |
| Gao et al,2018 | * | * | * | * | ** | * | * |  | 8 |
| Schraw et al,2017 | * | * | * | * | ** |  | * |  | 7 |
| Bailey et al,2017 | * | * | * | * | * | * | * |  | 7 |
| Mohammadian et al,2017 | * | * | * | * | * | * | * |  | 7 |
| Rios et al,2016 | * | * | * | * | * | ** | * |  | 8 |
| Greenop et al,2015 | * | * |  |  | * | * | * |  | 5 |
| Rudant et al,2015 | * | * | * | * | * | ** | * | * | 9 |
| Kucukcongar et al,2015 | * | * |  | * | * | * | * |  | 6 |
| Heck et al,2015 | * | * | * |  | * | * | * |  | 6 |
| Ajrouche et al,2015 | * | * | * | * | * | * | * |  | 7 |
| Schraw et al,2014 | * | * | * | * | * |  | * |  | 6 |
| Lupo et al,2014 | * | * |  |  | * | * | * |  | 5 |
| Diamantaras et al,2013 | * |  |  |  | * | * | * |  | 4 |
| Urayama et al,2012 | * |  |  |  | * | * | * |  | 4 |
| Crouch et al,2012 | * | * | * | * | ** | * | * |  | 8 |
| Waly et al,2011 | * | * | * | * | * |  | * | * | 7 |
| Rudant et al,2011 | * | * | * | * | ** |  | * | * | 8 |
| Ortega-Garcia et al,2008 | * | * | * | * | * | * | * |  | 7 |
| MacArthur et al,2008 | * | * | * |  | ** | * | * | * | 8 |
| Bener et al,2008 | * | * | * | * | ** |  | * |  | 7 |
| Harding et al,2007 | * | * | * |  | * |  | * |  | 5 |
| Altinkaynak et al,2006 | * |  | * | * | * |  | * |  | 5 |
| Saddlemire et al,2006 | * | * | * |  | ** | * | * |  | 7 |
| Kwan et al,2005 | * | * | * |  | ** |  | * | * | 7 |
| Jourdan et al,2004 | * | * | * |  | ** |  | * | * | 7 |
| Lancashire et al,2003 | * |  | * | * | * |  | * | * | 6 |
| Perrillat et al,2002 | * |  |  | * | ** |  | * |  | 5 |
| Murray et al,2002 | * | * |  |  | * | * | * |  | 5 |
| Schuz et al,2001 | * | * | * |  | * | * | * |  | 6 |
| Hardell et al,2001 | * | * | * | * | ** | * | * | * | 9 |
| Bener et al,2001 | * | * | * | * | ** | ** | * |  | 9 |
| Beral et al,2001 | * | * | * |  | * |  | * |  | 5 |
| Infante-Rivard et al,2000 | * | * | * | * | ** |  | * | * | 8 |
| Smulevich et al,1999 | * | * | * |  | ** | * | * | * | 8 |
| Shu et al,1999 | * | * | * |  | * |  | * | * | 6 |
| Schuz et al, 1999 | * | * | * | * | ** |  | * |  | 7 |
| Petridou et al,1997 | * | * | * |  | * |  | * |  | 5 |
| Shu, X. O et al,1995 | * | * |  |  | * | * | * |  | 5 |
| Shu, X. et al,1995 | * | * | * | * | ** |  | * | * | 8 |
| Magnani et al,1988 | * | * |  |  | * |  | * |  | 4 |
| Davis et al,1988 | * | * | * |  | * | * | * |  | 6 |
| Hartley et al,1988 | * | * | * |  | * |  | * |  | 5 |
| van et al,1988 | * | * | * |  | ** | * | * |  | 7 |
